# Short- and long-term mortality in patients with urosepsis caused by *Escherichia coli* susceptible and resistant to 3rd generation cephalosporins

**DOI:** 10.1186/s12879-022-07538-5

**Published:** 2022-06-24

**Authors:** Milena Tocut, Iris Zohar, Orna Schwartz, Orit Yossepowitch, Yasmin Maor

**Affiliations:** 1 Department of Medicine C, Wolfson Medical Center, Holon, Israel; 2 Infectious Disease Unit, Wolfson Medical Center, 62 Halochamim Street, 58100 Holon, Israel; 3 Sackler Faculty of Medicine, Tel-Aviv University, Tel-Aviv, Israel; 4 Microbiology and Immunology Laboratory Wolfson Medical Center, Holon, Israel

**Keywords:** Escherichia coli, ESBL, Resistance, Mortality

## Abstract

**Background:**

The aim of this study was to compare short- and long-term mortality among patients with urosepsis caused by *Escherichia coli* susceptibile (EC-SC) and resistant (EC-RC) to 3rd generation cephalosporins.

**Methods:**

A retrospective cohort study that included all patients with *E. coli* urosepsis admitted to a 700-bed hospital from January 2014 until December 2019. Mortality up to 30 days, 6 months and 1 year was assessed using logistic multivariate regression analysis and Cox regression analysis.

**Results:**

A total of 313 adult were included, 195 with EC-SC and 118 patients with EC-RC. 205 were females (74%), mean age was 79 (SD 12) years. Mean Charlson score was 4.93 (SD 2.18) in the EC-SC group and 5.74 (SD 1.92) in the EC-RC group. Appropriate empiric antibiotic therapy was initiated in 245 (78.3%) patients, 100% in the EC-SC group but only 42.5% in the EC-RC group. 30-day mortality occurred in 12 (6.3%) of EC-SC group and 15 (12.7%) in the EC-RC group. Factors independently associated with 30-day mortality were Charlson score, Pitt bacteremia score, fever upon admission and infection with a EC-RC. Appropriate antibiotic therapy was not independently associated with 30-day mortality. Differences in mortality between groups remained significant one year after the infection and were significantly associated with the Charlson co-morbidity score.

**Conclusions:**

Mortality in patients with urosepsis due to *E. coli* is highly affected by age and comorbidities. Although mortality was higher in the EC-RC group, we could not demonstrate an association with inappropriate empirical antibiotic treatment. Mortality remained higher at 6 months and 1 year long after the infection resolved but was associated mainly with co-morbidity.

## Introduction

Urinary tract infection (UTI) is one of the most common bacterial infections acquired both in the community and in hospitalized patients. Gram-negative Enterobacteriaceae including *Escherichia coli, Klebsiella pneumonia* and *Proteus mirabilis*, among others, cause most infections. Of these, *E. coli* is the most common pathogen accounting for 75–90% of urinary isolates [1]. Other less common pathogens include *Pseudomonas aeruginosa*, *Enterococcus* spp. and *Staphylococcus* spp. [1].

Over the past few decades, resistant Gram-negative bacteria have emerged, resulting in an increased burden on the health care system. Enterobacteriaceae resistance is mainly conferred by extended spectrum β-lactamases (ESBL) which are plasmid mediated enzymes capable of hydrolyzing the amide bond in the ß -lactam ring of antibiotics, resulting in multiple drug resistance (MDR) to earlier generations of penicillins and most cephalosporins [2].

In the past, according to the Clinical and Laboratory Standards Institute (CLSI), ESBL producing bacteria were identified by complicated phenotypic testing. As of 2010, laboratories moved to defining extended resistance according to the minimum inhibitory concentration (MIC) of Enterobacteriaceae to ceftriaxone. It is generallly accepted that a ceftriaxone MIC of 1 μg/ml or greater is ideal for detecting ESBL producing bacteria [3–5] but this method oversetimates ESBL cases as some bacteria have other mechanisms of resistance [6, 7].

UTI with *E. coli* is a common source of bacteremia [8]. Infections with ESBL organisms are associated with increased length of hospital stay, worse outcomes and increased mortality [9]. They are also associated with increased health costs [10].

The term "urosepsis" refers to an infection that originates in the urogenital system. About 30% of sepsis cases originate from the urinary system and are mainly caused by obstruction of the urinary tract by kidney stones, enlarged prostate in men, and malignancies [11].

Before starting empiric antibiotic therapy, the patient's risk factors for sepsis with *E.coli* resistant to 3rd generation cephalosporins should be evaluated and the empricial treatment chosen accordingly. Antibiotics choice is adjusted once the antibiogram is available [12].

Urosepsis is a serious and life-threatening illness. In 5% of the cases, the patients progress to sepsis, multi-system organ failure and septic shock [13]. The mortality rate in these patients can reach 40%. When the bacteria causing the infection is resistant to 3^rd^ generation cephalosporins, there is a higher chance of administering inappropriate therapy that may adversely affect outcomes.

The study objective was to compare the 30-day 6-months and 1 year mortality rate among patients with sepsis-derived urinary tract infection caused by *E. coli* susceptible to 3rd generation cephalosporins (EC-C) versus *E. coli* resistant to 3rd generation cephalosporins (EC-RC) and to assess the impact of resistance on outcomes. The study was approved by E. Wolfson Internal Review Board (0035-20-WOMC) and we received a waiver from informed consent.

## Methods

This was a retrospective study involving patients who were admitted to Wolfson Medical Center from 2014 to 2019 (January 2014 until December 2019). We received the list of all patients during this period with positive *E. coli* urine and blood cultures from the hospital’s microbiology laboratory database. Data was collected by accessing the patients' medical files through the hospital’s computer programs. Mortality up to 30 days, 60 days, 180 days, and 1 year was assessed by the mortality registry update, received monthly by the hospital's registry.

To be included patients had to have a positive blood culture with *E. coli* and a positive urine culture up to 48 h before or after the time blood culture was taken and to have a diagnosis of urosepsis in their medical file. Patients with an alternative reason for sepsis were excluded. We also excluded patients who died in the first 24 h after taking the blood culture as death may not be related to antibiotic resistance.

A predefined tabular questionnaire was used to complete data collection. The questionnaire included socio-demographic details, patient comorbidities, length of hospital stay, risk factors for urosepsis, risk factors for antibiotic resistance, hospital course, antibiotic treatment, and outcome. The Charlson score [14] was used to assess co-morbidity. We also used the Pitt bacteremia score (PBS) [15] to predict patients' outcomes.

Empiric antibiotic therapy was defined as treatment given for the first 72 h from taking the culture. Specific therapy was defined as antibiotic treatment given more than 72 h after the culture was taken. Appropriate therapy was defined as an antibiotic that the *E. coli* isolate was susceptible according to the microbiology laboratory tests (disk diffusion or E test or VITEK according to CLSI guidelines). If the patient received at least 24 h of appropriate treatment in the first 72 h treatment was considered appropriate.

Sensitivity to ceftriaxone as a representative of 3rd generation cephalosporines was defined as an MIC ≤ 1 µg/ml or disk zone diameter ≥ 23 mm. None of the patients received ceftazidime. During the study period the treatment of choice for patients presenting with urosepsis to the emergency department and considered to be low risk for having an infection with resistant pathogens (ESBL, Enterobacteriaceae with resistance to 3rd generation cephalosporines, or other resistant bacteria) was ceftriaxone or gentamicin. For patients with suspected resistant pathogens the protocol recommends amikacin, or piperacillin-tazobactam, or ertapenem.

### Statistics

Data were stored on an Excel spreadsheet (Microsoft, USA) and analysed using SPSS software v25 (IBM Inc.). Simple descriptive statistics, including means, medians, standard deviations (SD), and interquartile ranges, were used to describe the population. P value < 0.05 was considered significant. Patients with *E. coli* bacteremia sensitive to ceftriaxone (EC-SC) were compared to patients with bacteremia due to *E. coli* resistant to ceftriaxone (EC-RC). Comparisons were performed using Chi square test for categorical variables and Student’s t-test for continuous variables. A backwards conditional logistic regression model was created where the dependent variable was mortality. Candidate variables were chosen from the variable list. To enter the model, the association with the dependent variable was set at P < 0.1. We also performed Kaplan Meier with Log Rank analysis and Cox regression analysis to assess survival at 30 days, 180 days and 1 year. Hazard ratio (HR) and 95% confidence intervals (CI) were computed.

## Results

We identified 322 adult patients admitted Wolfson Medical Center over a period of 6years (2014–2019) with *E. coli* bacteraemia that fitted the inclusion criteria. Nine patients were excluded because they died in the first 24 h after taking the first blood culture thus the study included 313 patients. All were hospitalized in internal wards. Of these, 195 (62.3%) patients with the diagnosis of urosepsis caused by *E. coli* susceptible to 3rd generation cephalosporins (EC-SC group) and 118 (37.7%) patients with urosepsis caused by *E. coli* resistant to 3rd generation cephalosporin (EC-RC group).

Patients' characteristics are presented in Table 1.

**Table 1:** Characteristics of patients with *E. coli* urosepsis

	All patients N = 313	EC-SR group N = 195 (62.3)	EC-RC group N = 118 (37.7)	P value
Age (years), mean (SD)	79 (12.0)	79 (12.8)	80 (10.7)	0.376
Sex (female) n (%)	199 (63.6)	136 (69.7)	63 (53.4)	0.004
Nursing care institution, n (%)	57 (18.2)	18 (9.2)	39 (33.1)	< 0.001
Charlson score, mean (SD)	5.24 (2.18)	4.93 (2.27)	5.74 (1.92)	0.001
DM, (%)	133 (42.5)	83 (42.6)	50 (42.4)	0.974
BPH, n (%)	43 (13.7)	21 (10.8)	22 (18.6)	0.050
CRF, n (%)	50 (16.0)	22 (11.3)	28 (23.7)	0.004
Nephrolithiasis, n (%)	26 (8.3)	15 (7.7)	11 (9.3)	0.613
Urinary malignancy, n (%)	8 (2.6)	3 (1.5)	5 (4.2)	0.159
Permanent urinary catheter, n (%)	16 (5.1)	6 (3.1)	10 (8.5)	0.036
Recent urinary tract manipulation, n (%)	4 (1.3)	2 (1.0)	2 (1.7)	0.634
History of urinary retention, n (%)	6 (1.9)	3 (1.5)	3 (2.5)	0.676
History of recurrent UTI, n (%)	23 (7.3)	9 (4.6)	14 (11.9)	0.017
Previous hospitalization with EC-SC urosepsis, n (%)	7 (2)	4 (2)	3 (3)	1.0
Previous hospitalization with RC Enterobacteriaceae urosepsis, n (%)	18 (5.8)	6 (3.1)	12 (10.2)	0.009
Outpatient antibiotic therapy for UTI in the past 3 months, n (%)	25 (8.0)	4 (2.1	21 (17.8)	< 0.001

Data is presented as mean (SD) or number (%)

EC-CS Group—patients with urosepsis caused by *E. coli* susceptible to 3^rd^ generation cephalosporins

EC-RC Group—patients with urosepsis caused by *E. coli* resistant to 3rd generations cephalosporins

*SD* standard deviation, *DM* diabetes mellitus, *BPH* benign prostate hypertrophy, *CRF* chronic renal failure, *UTI* urinary tract infection, *S/P* status post

The cohort consisted of 117 males (36%) and 205 females (74%). The mean age of the patients was 79 (SD 12) years with a mean hospitalization stay of 11 (SD 9) days. Patients' characteristics are presented in Table 1. In the EC-RC group patients were more debilitated, a third resided in nursing homes and the mean Charlson score was 5.74 vs. the EC-SC group, where only 9.1% resided in nursing homes, and the mean Charlson score was 4.93. Of note, benign prostatic hypertrophy (BPH) was more common in the EC-RC group as was a permanent urinary catheter. Patients in the EC-RC group had a higher rate of recurrent urinary tract infections.

**Fig. 1:**
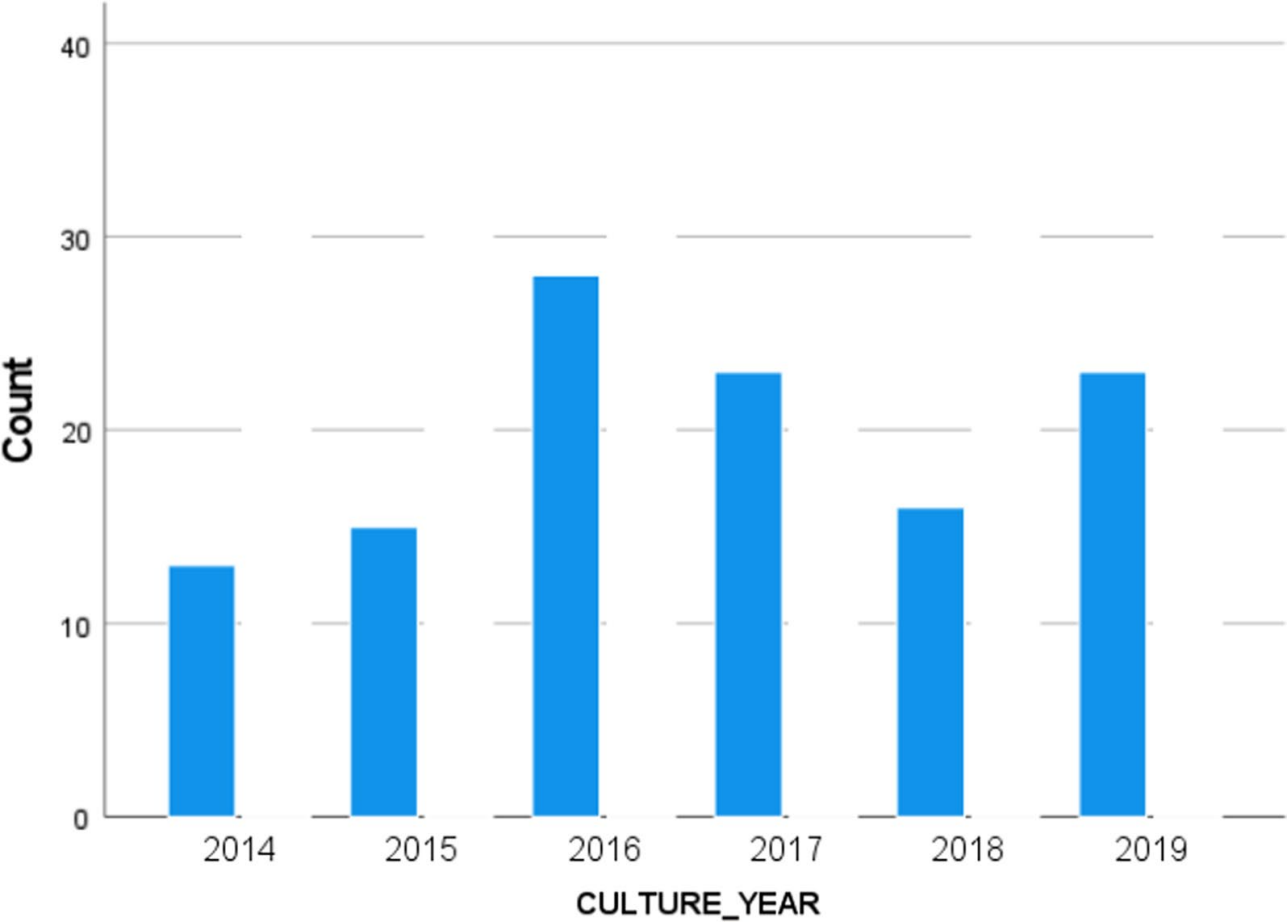
Distribution of EC-RC by year. Distribution is presented as absolute cases per year

The distribution of EC-RC by year can be seen in Fig. 1. The rate of resistant pathogens peaked in 2016 declined and then slightly increased again in 2019.

Table 2. describes the vitals and laboratory blood test at the day of the first positive culture. Patients in the EC-RC group had higher white blood cell (WBC) counts, and more positive blood cultures (more blood cultures were taken in this group due to a loer response rate to treatment). The Pitt bacteraemia score was similar in both groups.

**Table 2:** Vitals and laboratory blood tests

	All patients N = 313	EC-SC group N = 195 (62.3)	EC-RC group N = 118 (37.7)	P value
Systemic temperature (°C), mean (SD)	38.23 (1.22)	38.28 (1.30)	38.14 (1.08)	0.346
WBC K/microL, mean (SD)	15.492 (7.614)	14.788 (7.113)	16.655 (8.278)	0.035
CRP mg/l, mean (SD)	16.56 (10.33)	16.67 (10.35)	16.35 (10.34)	0.793
Albumin g/dl), mean (SD)	3.07 (0.63)	3.11 (0.47)	3.02 (0.83)	0.232
ARF, Cr > 1.3, n (%)	152 (48.6)	89 (45.6)	63 (53.4)	0.184
PBS, mean (SD)	0.79 (1.22)	0.77 (1.22)	0.81 (1.23)	0.756
Number of *E. coli* positive hemocultures, mean (SD)	1.29 (0.62)	1.13 (0.41)	1.55 (0.80)	< 0.001

Data is presented as mean (SD) or number (%)

EC-SC Group—patients with urosepsis caused by *E. coli* susceptible to 3^rd^ generation cephalosporins

EC-RC Group—patients with urosepsis caused by *E. coli* resistant to 3rd generation cephalosporins

*SD* standard deviation, *WBC* white blood cells, *CR* C reactive protein, *ARF* acute renal failure, *Cr* creatinine, *PBS* Pitt bacteremia score

Patients in the EC-RC group received longer antibiotic courses [8.20 (SD 2.80) vs. 7.40 (2.16) days, P = 0.005], as presented in Table 3. Appropriate empiric antibiotic therapy was initiated in 245 patients (78.3%), all patients in the EC-SC group but only 50 patients (42.4%) in the EC-RC group. Inappropriate treatment was mostly related to treatment with all classes of cephalosporines (67 cases, of these 66 cases received ceftriaxone), gentamicin (one case), ciprofloxacin (two cases), trimethoprim-sulfamethoxazole (one case) and piperacillin-tazobactam (one case). All isolates were sensitive to amikacin. Only two patients failed to receive appropriate antibiotic therapy after 72 h once the bacteria were identified and antibiotic susceptibilities were available.

**Table 3:** Hospital course and outcomes of patients with *E. coli* urosepsis

	All patients N = 313	EC-SC group N = 195 (62.3)	EC-RC group N = 118 (37.7)	P value
Antibiotic administration (days), mean (SD)	7.70 (2.45)	7.40 (2.16)	8.20 (2.80)	0.005
Appropriate empiric antibiotic therapy, n (%)	245 (78.3)	195 (100)	50 (42.4)	< 0.0001
Septic shock, n (%)	15 (4.8)	5 (2.6)	10 (8.5)	0.018
Surgical drainage, n (%)	7 (2.2)	3 (2.5)	4 (2.1)	0.776
Mechanical ventilation, n (%)	16 (5.1)	7 (3.6)	9 (7.6)	0.116
Length of hospital stay (days), mean, (SD)	11.46 (8.50)	9.29 (6.40)	15.04 (10.19)	< 0.0001
Mortality within 30 days, n (%)	27 (8.6)	11 (5.6)	16 (13.6)	0.016
Mortality within 60 days, n (%)	37 (11.8)	14 (7.2)	23 (19.5)	0.001
Mortality within 180 days, n (%)	50 (16)	22 (11.3)	28 (23.7)	0.004
Mortality within 1 year, n (%)	55 (17.6)	25 (12.8)	30 (25.4)	0.005

Data is presented as mean (SD) or number (%)

EC-SC Group—patients with urosepsis caused by *E. coli* susceptible to 3rd generation cephalosporins

EC-RC Group—patients with urosepsis caused by *E. coli* resistant to 3rd generations cephalosporins

*SD* standard deviation

Urosepsis complications were more common in the EC-RC group. Septic shock occurred more commonly in the EC-RC group. There was no difference between the groups in the need for surgical drainage or mechanical ventilation.

Thirty-day mortality occurred in 27 (8.6%) patients, 11 (5.6%) in the EC-SC group and 16 (13.6%) in the EC-RC group, P = 0.016. 60-day mortality increased to 37 (11.8%) patients [14 (7.2%) in EC-SC group vs. 23 (19.5%) in the EC-RC group, P = 0.001], 180-day mortality increased to 50 (16%) [22 (11.3%) vs 28 (23.7%), respectively, P = 0.004]. One year mortality occurred in 55 (17.6%) patients, 25 (12.8%) in the CS group and 30 (25.4%) in the EC-RC group (P = 0.005). In a logistic regression, predictors of 30-day mortality were the Charlson score, the Pitt bacteremia score, and infection with a *E. coli* resistant to 3rd generation cephalosporins. Appropriate antibiotic treatment was not significantly associated with 30-day mortality (Table 4). Six months mortality remained significantly higher in the EC-RC group and more than doubled in this group compared to the 30 days death rate. Increase in mortality at 1 year compared to 6 months was much smaller.

**Table 4:** Logistic regression of 30 days mortality

	Univariate analysis			Multivariate analysis		
	P-value	OR	95% CI	P-value	OR	95% CI
Gender (female)	0.086	0.499	0.226–1.103			
Permanent Nursing Home stay	0.038	2.479	1.051–5.848			
Charlson comorbidity index score*	< 0.001	1.395	1.170–1.662	< 0.001	1.437	1.172–1.763
History of urinary retention	0.489	2.162	0.243–19.204	–	–	–
PBS*	< 0.001	1.625	1.259–2.099	< 0.001	1.644	1.151–7.29
Fever upon admission*	0.209	0.831	0.622–1.110			
CRP*	0.028	1.043	1.005–1.083	–	–	–
ARF*	0.054	2.269	0.986–5.219	–	–	–
EC-CR*	0.019	2.624	1.173–5.868	0.024	2.885	1.151–7.229
Appropriate empirical antibiotic treatment	0.131	0.520	0.222–1.216	–	–	–
Constant	–	–	–	< 0.001	0.004	–

Nagelkerke R square 0.239

*PBS* Pitt bacteremia score, *CRP* C reactive protein, *ARF* acute renal failure, *EC-CR* *E. coli* resistant to 3rd generation cephalosporins, *OR* odds ratio, *CI* confidence interval

^*^Variables that were entered into the multivariate logistic regression

**Fig. 2:**
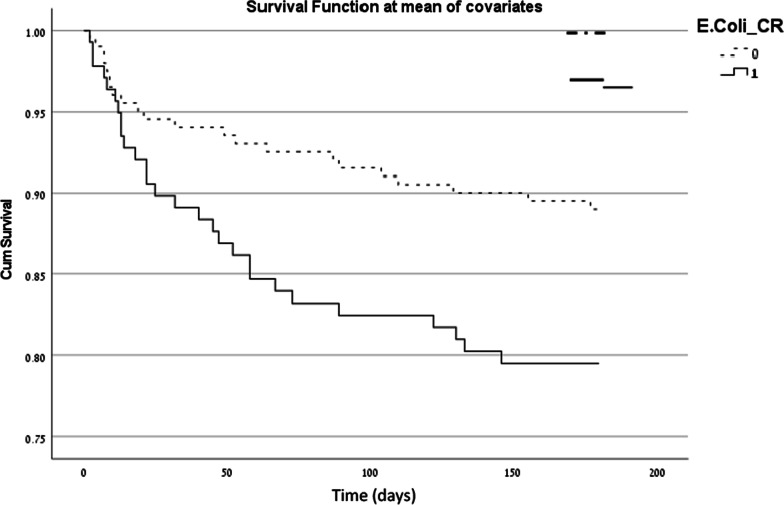
Cox regression of survival at 6 months. Time (days)—time to death at 180 days. Candidate variables were age, gender and Charlson score. Difference in mortality was significant between the two groups (Kaplan Meier log rank P = 0.003). In the Cox survival analyses Charlson score was significantly associated with 180-day mortality (HR 1.168 95% CI 1.022–1.335]

**Fig. 3:**
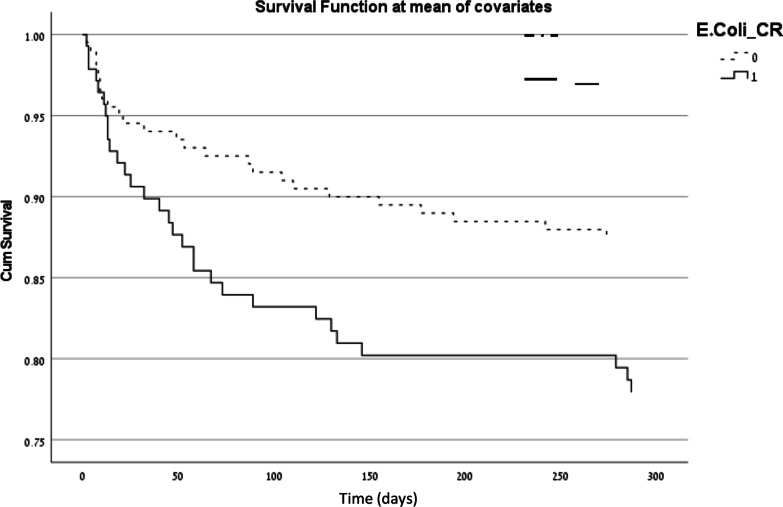
Cox regression of survival at one year. Time (days)—time to death at 1-year. Candidate variables were age, gender and Charlson score. Results were similar at one year with mortality remaining significantly higher in EC-RC group (Kaplan Meier log rank P = 0.004). In the Cox survival analyses Charlson score was significantly associated with 1-year mortality (HR 1.162 95% CI 1.024–1.320]

In a logistic regression, predictors of 1 year mortality were the Charlson score, CRP levels, acute renal failure, and infection with *E. coli* resistant to 3^rd^ generation cephalosporins. (Table 5). Figures 2, and 3 depict the mortality over time comparing patients with EC-SC and patients with EC-RC in a Cox regression model adjusted for age gender, and Charlson score. 30 day mortality was significantly higher in the EC-RC group (Kaplan Meier log rank P = 0.017). In the Cox survival analyses Charlson score was significantly associated with 30-day mortality [hazard ratio (HR) 1.318 95% confidence interval (CI) 1.121–1.548]. At 180 days, mortality remained significantly higher in EC-RC group (Fig. 2) (Kaplan Meier log rank P = 0.003). In the Cox survival analyses Charlson score was significantly associated with 180-day mortality (HR 1.168 95% CI 1.022–1.335]. Results were similar at 1 year with mortality remaining significantly higher in EC-RC group (Fig. 3) (Kaplan Meyer log rank P = 0.004). In the Cox survival analyses Charlson score was significantly associated with 1-year mortality (HR 1.162 95% CI 1.024–1.320].

**Table 5:** Logistic regression of one year mortality

	Univariate analysis			Multivariate analysis		
	P-value	OR	95% CI	P-value	OR	95% CI
Gender (female)*	0.127	0.632	0.350–1.140	–	–	–
Permanent Nursing Home stay	0.128	1.707	0.857–3.402	–	–	–
Charlson comorbidity index score*	0.001	1.245	1.089–1.424	0.026	1.185	1.021–1.376
History of urinary retention	0.320	2.396	0.428–13.422	–	–	–
PBS*	0.104	1.188	0.965–1.461	–	–	–
Fever upon admission	0.172	0.854	0.680–1.071	–	–	–
CRP*	0.033	1.031	1.003–1.061	0.067	1.028	0.998–1.059
ARF*	0.015	2.109	1.156–3.849	0.082	1.786	0.929–3.434
EC-RC*	0.005	2.318	1.285–4.181	0.005	2.455	1.306–4.613
Appropriate empirical antibiotic treatment*	0.013	0.444	0.235–0.840	–	–	–
Constant	–	–	–	< 0.0001	0.024	–

Nagelkerke R square 0.134

*PBS* Pitt bacteremia score, *CRP* C reactive protein, *ARF* acute renal failure, *EC-RC* *E. coli* resistant to 3rd generation cephalosporins, *OR* odds ratio, *CI* confidence interval

^*^Variables that were entered into the multivariate logistic regression

## Discussion

In this study, we demonstrated that patients with EC-SC urosepsis had a more indolent course of disease with better outcomes and mortality rate than patients with EC-RC urosepsis. Patients with *E. coli* urosepsis were elderly with a high Charlson score. Mortality rate in the year after the infection was high in both groups but peaked to 25.4% in the EC-RC group. After adjusting for age and co-morbidities the difference in mortality was not significant.

Following the increasing prevalence of resistant Enterobactereceae causing urosepsis several studies tried to identify and deremine risk factors for acquired infections with resistant pathogens and in particulatr Enterobacterecae resistant to 3rd generation cephalopsporins (formerly described as ESBL). Several risk factors were suggestedincluding older age, reccurent UTI, previous ESBL infections, use of third generation cephalosporins or fluoroquinolones in the previous three months, prior hospitalization, recent transfer from another health care institution, Charlson comorbidity score ≥ 4, and recent urinary catheterization or procedure [15–20].

Risk factors found to be significantly associated with EC-RC urosepsis in this study were gender, permanent nursing institution patients, a higher Charlson score, BPH, chronic renal failure, a history of recurrent UTIs, previous outpatient antibiotic therapy for UTI, and previous hospitalizations with urosepsis due to Enterobacteriaceae resistant to 3rd generation cephalosporins.

Urosepsis is the second most common cause of septic shock and is more prevalent in men [13]. The EC-RC group consisted predominantly of femaes (63%), in comparison to the EC-SC group which did not differ numerically between genders. Females may be more prone to reccurent UTIs, which further increases the risk for antibiotic resistant *E. coli*. [21].

One study showed that patients with *E. coli* ESBL urosepsis had milder disease manifestation than patients with *E. coli* susceptible to 3rd generation cephalosporins urosepsis [22] and most studies suggested that the in hospital and 30-day mortality was not affected by 3rd generation cephalosporin resistance [23, 24]. In this study 30-day mortality was significantly higher in the EC-RC group, and more than half of the patients in the EC-RC group received empirical inappropriate antibiotic therapy. Differences in mortality between the two groups persisted up to 1 year after the bacteremia. In a multivariate logistic regression inappropriate antibiotic treatment was not significantly associated with 30-day mortality. Charlson score was significantly associated with mortality at 180 days and 1 year after the infection. Although differences between the two groups regarding mortality remained up to 1 year after the infection most of the difference between the groups occurred in the first month after the infection. Two meta analyses showed that high mortality rates associated with ESBL bacteremia were thought to result from delayed appropriate antibiotic therapy rather than the high virulence of ESBL strains [7, 25]. As patients suffering from urosepsis are elderly with many co-morbidities it may be difficult to separate these factors from the effect of resistant pathogens and the effect of inappropriate treatment, and to correctly identify all confounders. Not many studies assessed long term outcomes of resistant *E. coli* infections. In a study from Sweden [26] there was no difference in mortality rates 1 year after the infection between patients with ESBL pathogens compared to patients without ESBL resistance. Patients in this cohort were 4–6 years younger than the patients in our study. Thirty-day mortality in our cohort was 8.6%. In the Swedish cohort mortality was higher and reached 15% [26], in a French cohort mortality was 9.5% [27], and in an American cohort between 8 and 12% [28]. Higher mortality rates have been observed when assessing other Enterobacteriaceae causing urosepsis such as *K. pneumonia*. These isolates have a higher chance of being resistant to 3^rd^ generation cephalosporins. In a report by Lee et al. [29] mortality from urosepsis due to *E. coli* was 7.8% versus 15% in patients with urosepsis due to *K. pneumonia*. Mortality is most probably related to the rate of resistant pathogens and the fact that in our cohort we did not have patients with *E. coli* resistant to carbapenems.

Most isolates in our study were sensitive to amikacin. We recently demonstrated that specific therapy with amikacin in patients not in septic shock and with creatinine clearance above 30 ml/min is safe and effective [30]. It was recently also shown that an institutional aminoglycoside-based regimen as empirical treatment of patients with pyelonephritis is safe and effective [31]. As drug resistance rates are rising both in the community and within hospitals [32, 33] it is important to choose correctly appropriate empirical regimens.

This study has several caveats. As data was gathered retrospectively information was limited to the patient’s follow up and physician’s notes. Therefore, information about past medical history of recurrent UTI and antibiotic therapy as outpatients may have been missed. We included only one medical center, thus resistant rates and patient population may differ between centers.

To conclude, mortality in patients with urosepsis due to *E. coli* is highly affected by age and comorbidities. Although mortality was higher in the EC-RC group, we could not demonstrate an association with inappropriate empirical antibiotic treatment. Mortality remained higher at 6 months and 1 year after the infections in the EC-RC group but was mostly related to differences in co-morbidities between patients with susceptible and resistant *E. coli*. Infection with EC-RC is associated with an increased death rate after the primary infections has resolved. Further study is warranted to better understand the factors affecting this increased death rate.

## Data Availability

Data can be obtained upon request from Dr. Yasmin maor.
